# 3-Self Behavior Modification Programs Base on the PROMISE Model for Clients at Metabolic Risk

**DOI:** 10.5539/gjhs.v4n1p204

**Published:** 2012-01-01

**Authors:** Ungsinun Intarakamhang

**Affiliations:** Behavioral Science Research Institute Srinakharinwirot University, Bangkok, Thailand E-mail: ungsinun@swu.ac.th

**Keywords:** CIPP Model, Behavior modification, Metabolic syndrome, Self-regulation, Self-care

## Abstract

The objectives of this mixed methods research were 1) to study effects of the health behavior modification program (HBMP) conducted under the principles of the PROMISE Model and the CIPP Model and 2) to compare the 3-self health behaviors and the biomedical indicators before with after the program completion. During the program, three sample groups including 30 program leaders, 30 commanders and 120 clients were assessed, and there were assessments taken on 4,649 volunteers who were at risk of metabolic syndrome before and after the program conducted in 17 hospitals. The collected data were analyzed by the t-test and the path analysis. The research instruments were questionnaires used for program evaluation, structuralized interview forms, and questionnaires used for 3-self health behavior assessment. The findings were as follows: 1) During the program, the assessment result deriving from comparing the overall opinions toward the program among the three sample groups showed no difference (F=2.219), 2) The program management factors based on the PROMISE Model (positive reinforcement, optimism, context, and process or activity provision) had an overall influence on the product or success of the HBMP (p< 0.05) with size effects at 0.37, 0.13, 0.31 and 0.88 respectively. All of the factors could predict the product of the program by 69%. 3) After participating in the program, the clients’ 3-self health behaviors (self-efficacy, self-regulation, and self-care) were significantly higher than those appeared before the participation (p< 0.05), and their biomedical indicators (BMI, blood pressure, waistline, blood glucose, lipid profiles, cholesterol, and HbA1c) were significantly lower than those measured before the program (p< 0.05).

## 1. Introduction

The National Health Security Office (NHSO) has underlined health problems associated with metabolic syndrome (MS) including obesity, high hypertension, diabetes, and stroke as such diseases cause illness to people worldwide: the U.S. (24% of the total population or 44% of adults aged over 50 years); Saudi Arabia (39.3%); Turkey (33%); Tehran, Iran (30%); and South Korea (14.2%) (Shila *et al.*, 2010). In Thailand, 36% of the people living in Bangkok suffered from MS. In addition, the mortality rate per 100,000 Thai people due to cardiovascular disease constantly increased from 48.58 persons in 2002 to be 55.29 persons in 2007. During the period, the mortality rate attributed to both cardiovascular disease and diabetes was as high as 85,000 people per year or 236 people per day. It was also found that 58% of people who survived a stroke remained permanently disabled and became a burden on the society due to high medical expenditures of long-term treatment (Health Research Network, 2009). Thus, health behaviors and MS are key indicators of having good quality of life (Wood, 2005). Promotion of health behaviors needs cooperations from all segments of the society in an effort to solve health problems for overall achievement and to yield substantial results to the public (Knezevic *et al.*, 2008; Knezevic, 2009; WHO, 2010). At present, the health behavior modification adhering to accurate psychological techniques and strict occupational ethics is approved to be sustainable prevention of health problems and the safest way for people in that it can reduce mortality rate and risk of medications (Innis, 1981; Watanabe *et al*, 1998). Many developed nations lay importance on the health problem prevention with the use of health behavior modification techniques that combine psychological theories with social cognitive learning (Bandura, 1986). The techniques are used to design activities that suit specific features of the at risk group so as to reduce risky health behaviors and reinforce preferred ones which consequently lessen occurrence of chronic diseases (Jones, Brockway & Atkinson, 1995). Behavior modification includes group activities and community relations which help reduce risk of obesity (Nilsen, 1996); it can also be achieved through sports and exercise (Larsen & Manderson, 2009). People of all ages and occupations can individually and collaboratively participate in behavior modification activities; however, such activities must rely on an understanding in psycho-behavioral science. Hence, the providers of health behavior modification must receive training on accurate modification techniques, methods, and procedures by the experienced, and there must be a regular follow-up in order to achieve the set goals (Elder, 1987; Leventhal & Linda, 1987; Reighard, 2010).

The plan to modify the 3-self health behaviors (self-efficacy, self-regulation, and self-care) of Thai people at risk of MS was developed by the NHSO, in collaboration with Srinakharinwirot University, on the basis of the PROMISE Model: P = Positive Reinforcement, R = Result-based Management, O = Optimism, M = Motivation, I = Individual or Client Center, and SE = Self-esteem (Ungsinun, 2009). In addition, in 2010 the Thai Public Health Ministry has launched the policy of health adjustment village with an aim to decrease the risk of cancer, high blood pressure, and cardiovascular disease by using key behavioral indicators which are an exercise program of at least three to five days a week and daily consumption of half a kilogram of fruits and vegetables (Health Education Division, 2011). This research was conducted with the cooperation of 100 program leaders from 17 hospitals in Bangkok Metropolis, amounted to 30 projects all together. After the participating leaders received a 4-day training in the techniques of 3-self health behavior modification in accordance with the PROMISE Model, they had to carry out ongoing health activities for 4,649 people who were at risk of MS for at least five times during five to seven months. Meanwhile, they were periodically supervised, monitored, and assessed by the researcher team.

## 2. Objectives of the research

The main purpose of this study were 1) evaluating the HBMP carried out by the participating hospitals, based on the CIPP Model and the 360 Degree Feedback, 2) examining effects of the health programs administered according to the PROMISE Model by focusing on the context, the input, and the process toward the product received from the programs, 3) comparing the 3-self health behaviors of the clients before participating the program with the ones occurred at the end of the program, and 4) making a comparison of the biomedical indicators (BMI, blood pressure, waistline, blood glucose, lipid profiles, cholesterol, and HbA1c) before and after the program.

## 3. Methods

### 3.1 Extent of evaluation

The evaluation research was conducted in line with the CIPP Model of Stufflebleam & Shinkfield (2007), as well as the Logic Model of Pankratz (2008).

### 3.2 Setting

The setting included 30 projects of 17 hospitals in Bangkok Metropolis which were funded by the NHSO in fiscal year 2010.

### 3.3 Samples

3.3.1 The data used in experimental research were 4,649 Thai clients aged over 15 years, living in Bangkok Metropolis, and proved to be patients or at risk of MS; obesity, diabetes, high hypertension, and stroke. The clients volunteered to participate in the health behavior modification program for 5 to 7 months.

3.3.2 The data used in the evaluation research were collected during the program from 30 program leaders, 30 commanders of the program leaders, and 120 clients.

### 3.4 Instruments

3.4.1 Structured interview forms were used to investigate the context, the input, the process, and the product of the HBMP.

3.4.2 Four-point rating scale questionnaires consisting of 40 questions were used to evaluate the program based on the CIPP Model. The item discrimination of the questionnaires was found to range between .478 to .733 with the Cronbach’s alpha coefficients of reliability at .873.

3.4.3 Four-point rating scale questionnaires containing 20 questions were used to evaluate the program administered under the PROMISE Model. The questionnaires were completed by the clients and the program leaders. The item discrimination of the questionnaires were between .509 to .754 with the reliability at .938.

3.4.4 Four-point rating scale Questionnaires with 17 questions were launched before and after the program inquiring about the 3-self health behaviors of the clients: 1) *Self-efficacy* involves the confidence in one’s ability to adjust health behaviors by oneself with patience and endeavor until achieving the set goal, the part’s reliability equaled .730, 2) *Self-regulation* is to observe one’s behaviors with changes in one’s health and to set a goal of having good health and a plan to achieve the goal by recording changes in health behaviors as well as reminding of ongoing action, the part’s reliability was at .800; 4.3) *Self-care* referred to one’s habit of having regular health check, searching for health knowledge, consuming proper food, exercising, and managing stress with an aim to live in good health. Behaving accordingly relied on a practice and a continuous process. The reliability was at .850.

3.4.5 Biomedical instruments comprising, as examples, measurements of BMI, blood pressure, waistline, blood glucose, lipid profiles, cholesterol, and HbA1c which were taken before and after the program.

### 3.5 Hypotheses

3.5.1 The result of the CIPP Model-based evaluation of the health program is at the good level and indicates the consistency in opinion among the groups of program leaders, clients, and commanders of the program leaders.

3.5.2 The factors of the program administration based on the PROMISE Model, namely context, input, and process has an impact on the product of the HBMP.

3.5.3 At the completion of the program, the clients’ self-efficacy, self-regulation, and self-care are higher than the beginning of the program.

3.5.4 The clients’ biomedical indicators (BMI, blood pressure, waistline, blood glucose, lipid profiles, cholesterol, and HbA1c) are better than before the program.

### 3.6 Statistical Analysis

3.6.1 To test Hypothesis 1, one-way ANOVA was used in comparing the consistency of the CIPP Model-based evaluation scores deriving from the three sample groups.

3.6.2 To test Hypothesis 2, the path analysis was used to analyze the quantitative data about the program administration and the activity provision based on the PROMISE Model during the program so as to study the effect size representing the influence of the factors including positive reinforcement, result-based management, optimism, motivation, individual or client center, self-esteem, context, input, and process that affected the product or success of the program.

3.6.3 To test Hypothesis 3 and 4, there were comparisons of the 3-self health behaviors and the biomedical indicators before and after the program with the use of the dependent t-test.

## 4. Results

*4.1* *As for* *general information* concerning *the clients*, most of them were female, totaling 3,241 persons (69.71% of the total); 1,197 of them aged between 50 to 59 (25.75%). 3,308 of the clients held an academic degree lower than a bachelor’s degree (71.16%). 1,734 clients were under the medical benefit scheme for civil servants or state enterprise employees, including their family members (37.30%). 1,545 clients faced the risk of obesity (33.23%), and 3,248 clients had the BMI higher than 23 kg/m^2^ (69.86%). 3,783 clients (81.37%) were very satisfied with the HBMP.

*4.2* *The* *outcome of the CIPP Model-based evaluation of the program* revealed that, generally, the evaluation scores of the clients, the program leaders, and the commanders of the program leaders were not diverse and found to be in the good level (the average scores=3.52, 3.47, 3.45 respectively) as show in Table 1.

**Table 1:** The comparison of the consistency of the evaluation scores among the clients, the program leaders, and the commanders of the program leaders using the ANOVA and the t-test.

Variables (scale 1 – 4)	clients(1) n =120		program leaders (2) n =30		commanders (3) n =30		F-test / (t-test)	Scheffe test		
	Mean	S.D	Mean	S.D	Mean	S.D		1-2	1-3	2-3
1.Context	3.54	.32	3.50	.33	3.48	.30	.478	.04	.06	.02
2.Input	3.45	.35	3.40	.32	3.39	.35	.550	.05	.06	.01
3.Process	3.51	.39	3.52	.34	3.13	.48	10.448**	-.01	.38*	.39*
4.Product	3.56	.54	3.38	.32	3.39	.39	2.415	.18	.17	-.01
5.Total	3.52	.33	3.45	.28	3.35	.32	3.219*	.07	.17*	.10
6.PROMISE	3.53	.37	3.58	.31	-	-	(t = .596)	-	-	-

* P-value < .05,

** P-value <.01

**Figure 1:**
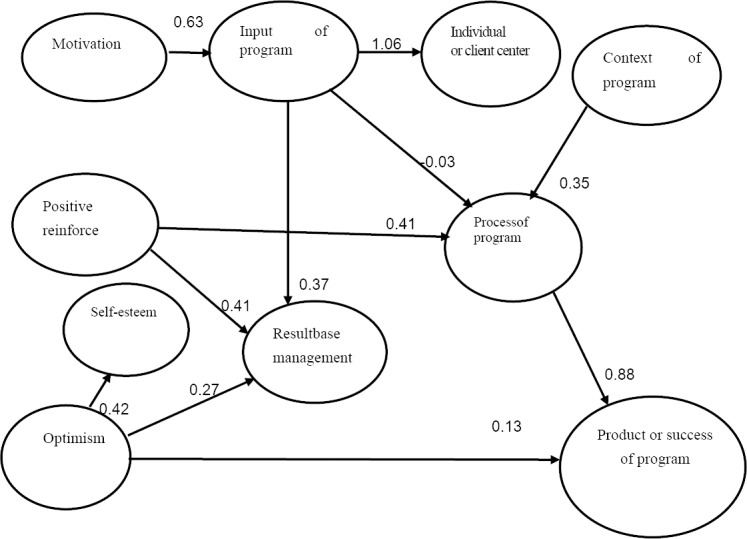
The casual correlation model and effect size of the product or success of the program

*4.3* *The results of the analysis on the impact of the key factors* on the product of HBMP carried out by the participating hospitals using the LISREL program showed that the factors concerning positive reinforcement, optimism, motivation, context, and input of program generally affected the product or success of the HBMP with effects at 0.37, 0.13, -0.02, 0.31, and -0.02 respectively, and the process was found to had direct effect on the product of the program with highest effect at 0.88 (χ^2^= 29.8; df =19; p-value = 0.15489; RMSEA = 0.051; RMR = 0.0082; CFI = 0.99; AGFI = 0.86; GFI = 0.95; CN = 142.10). The relationship between the casual factors and the product of the program was displayed in Figure 1.

*4.4* *After finishing the HBMP*, the client’s self-efficacy, self-regulation, self-care, as well as biomedical indicator scores improved as shown in Table 2.

**Table 2:** Comparison of 3-self behavior and biomedical indicator scores between before and after participating in the program

Health risk indicators	n	Before		After		MD.	t
		X̄	S. D.	X̄	S.D.		
Self-Efficacy	4,649	13.59	2.89	15.72	2.74	-2.12	52.850*
Self-Regulation	4,649	13.44	3.11	15.75	2.74	-2.32	56.378*
Self-Care	4,649	18.48	3.75	21.28	3.48	2.93	54.991*
BMI	3,832	26.39	4.61	25.95	4.51	.44	19.896*
Systolic BP	2,754	127.73	16.88	124.51	14.60	3.21	8.115*
Diastolic BP	2,753	78.95	10.76	77.15	12.65	1.80	8.115*
Waistline	918	31.57	3.99	31.00	3.91	.57	6.858*
Waist hip ratio	100	.8761	.0642	.8673	.0652	.0088	1.701
Fasting blood sugar	712	105.59	38.95	103.26	39.32	2.33	2.296*
Lipid profiles	100	31.25	8.91	30.24	9.64	1.01	2.335*
Cholesterol	355	236.62	28.57	205.26	36.06	31.36	12.810*
HbA1c	144	9.08	2.28	8.01	1.67	1.06	7.189*

* P-value < .05

*4.5* *Key elements to successful HBMP* were that the clients displayed attention and willingness toward the program by attending the health activities every time they were conducted. In addition, the clients were given positive reinforcement, optimism, motivation by the staff, as well as support by the commanders. Besides, the activities were diversified and mainly focused on the service receivers.

## 5. Discussion

*5.1 Having tested Hypothesis 1*, the evaluation scores deriving from the clients, the program leaders, and the commanders of the program leaders were found consistent for many reasons. First, all of them perceived the necessity of the program and wanted to respond to the hospital’s policies. Next, all people that at risk were permitted to be part of the health care program; they were allowed to set their own goals and run activities by themselves in order to solve their health problems. Finally, all of the three groups showed hospitality and friendliness to one another; such phenomenon conformed to the concept of Bandura (1986) on behavior modification in that success of behavior modification derives from willingness of the individuals whose behaviors are to be adjusted and the others involved.

*5.2 According to Hypothesis 2*, the factors including positive reinforcement, optimism, context, and process of HBMP were found to have an impact on the product or success of the program at the 0.05 level of significance with effects at 0.37, 0.13, 0.31, and 0.88 respectively, and they were able to predict the product or success of the program by 69%. This explained that the staff administered the program by relying on ‘positive reinforcement’ of the PROMISE Model. In such doing, when the clients’ behaviors continually changed to the target ones, the clients were convinced that they would receive awards and acceptance for their efforts. This finding was in line with Burnet *et al*. (2002) who synthesized the research on the development of behavior modification programs for diabetes type 2 prevention and found that many research studies succeeded in implementing positive reinforcement into health programs and the effects of an optimistic program on a sense of happiness in life of patients with high blood pressure revealed that the experimental group were happier than the controlled group at the 0.01 level of significance.

*5.3 Regarding the tests of Hypothesis 3 and 4*, it was found that the clients’ 3-self behaviors and biomedical indicators improved. This was because the PROMISE Model-based program administration encouraged the clients to play an important role in designing and doing health behavior modification programs by themselves. In addition, they were allowed to be a program initiator and an activity administer till the end of the program; as a result, the clients developed the feeling of being the program owner. Besides, there was use of positive psychological techniques to build the clients’ motivation resulting in the demand for activity attendance. These findings agreed with that of Grey *et al*. (2004) who conducted a pilot study testing the effectiveness of the prevention program conducted for 41 teenagers who were at risk of diabetes type 2 in two middle schools in Connecticut for a period of 12 months. The students of the experimental group were permitted to be part of activities in order to adjust their own behaviors. The activity provider only persuaded the students by talking to them, and invited their parents to take care of the students’ nutrition and to be part of their physical activities. The children were given supports and advices via telephone. Grey el al.’s research explained that, in general, the students of the experimental group and their parents gained more health knowledge and developed better health behaviors. Also, the result of this research was consistent with that of Thitima (2006) who investigated the 10-week behavior adjustment regarding weight loss in the overweight people based on Bandura’s self-regulation concept, combining with social supports which included knowledge providing, self-observation processing, decision making processing, exercise practicing, stimulating, and morale support and advice giving. The overweight clients in the experimental group were found to have higher frequency in weight loss, lose more weight, and consequently have less weight than the controlled group at the 0.01 level of significance. Achara (2008; cited in Ungsinun, 2009) also suggested that motivation could cause an ongoing behavior which occurred for a particular purpose. The fact that the staff of the participating hospitals brought motivation reinforcement into the health projects resulting in the occurrence of self-care behaviors of the clients. This was considered to conform with the thought of Barofsky (1978; cited in Orasa, 1999) explaining that self-care was a person’s intentional action with some reason concerning health; therefore, without motivation or intention regarding self-care, self-care behaviors would not occur. Moreover, the research of Suwannee (2002) that examined effects of self-control skill development of students on avoidance of snack consumption by using the concept of motivation reinforcement found that after the experiment the experimental group developed the behaviors of snack avoidance more than before the experiment and than that of the controlled group at the 0.05 level of significance. In the same way, Mananya (2005) studied effects of individual consultation toward self-control concerning weight loss in a group of 20 volunteers during an eight-week period. There were six stages of self-control which were relationship building, risky behaviors examining, weight estimating, planning and self-promising, plan implementing, and consultation ending. The research results revealed that the experimental group had significantly more improvements in behaviors of noticing and recording their eating and exercising than the controlled group and than before the experiment at 0.01.

## 6. Conclusion

The health behavior modification program for people at risk of metabolic syndrome conducted on the PROMISE Model was able to cause increases in the clients’ 3-self behaviors and improve their biomedical indicators.

## 7. Recommendations for future research

7.1 Commanders should encourage the staff by giving them rewards and positive reinforcement.

7.2 Clients with similar ages or health problems should be grouped together so that it will be more convenient for developing motivation strategies.

7.3 Regarding the features of activities, the clients must voluntarily participate in the program provided, and they should be allowed to create and adjust activities that individually suit their behaviors. The activities may be divided into three parts: games of cognitive adjustment and thinking change (30%); knowledge of 3-E (energy diet, exercise, and emotional management) (20%); and skill practices of the 3-self behaviors and 3-E (50%). Group activities should be carried out under the regulations firmly established in order to discipline the clients, and all groups of people should be welcomed to participate in the activities. To be more sustainable, these health programs should be promoted as routine work.
